# Rare *PANK2* variants and pantothenate-kinase-associated neurodegeneration in the Dominican Republic

**DOI:** 10.1093/braincomms/fcaf286

**Published:** 2025-08-04

**Authors:** Badri N Vardarajan, Pedro Sanchez Roa, Christine Y Kim, Peter Stoeter, Diones Rivera Mejia, Alexander Houck, Amanda Chan, Dolly Reyes-Dumeyer, Angel Piriz, Robert Fee, Francisco Blanco-Abinader, Francisco A Roedan, Elizabeth Rice, Samantha Christenson, Rebecca Chiu, Tamil I Gunasekaran, Rafael A Lantigua, Clifton Dalgard, Serge Przedborski, Richard Mayeux

**Affiliations:** Department of Neurology, Vagelos College of Physicians and Surgeons, Columbia University, and the New York Presbyterian Hospital, New York, NY 10032, USA; The Gertrude H. Sergievsky Center, Vagelos College of Physicians and Surgeons, Columbia University, and the New York Presbyterian Hospital, New York, NY 10032, USA; Department of Neurology, Centro Medico de Diabetes, Obesidad y Especialidades (CEMDOE), Santo Domingo, 10135, Dominican Republic; Department of Neurology, Vagelos College of Physicians and Surgeons, Columbia University, and the New York Presbyterian Hospital, New York, NY 10032, USA; Department of Radiology, CEDIMAT, Plaza de la Salud, Santo Domingo, 10514, Dominican Republic; Department of Neurosurgery, CEDIMAT, Plaza de la Salud, Santo Domingo, 10514, Dominican Republic; Department of Neurosurgery, Universidad Pedro Henríquez Urena, Santo Domingo, 1423, Dominican Republic; Department of Neurology, Vagelos College of Physicians and Surgeons, Columbia University, and the New York Presbyterian Hospital, New York, NY 10032, USA; Department of Neurology, Vagelos College of Physicians and Surgeons, Columbia University, and the New York Presbyterian Hospital, New York, NY 10032, USA; The Gertrude H. Sergievsky Center, Vagelos College of Physicians and Surgeons, Columbia University, and the New York Presbyterian Hospital, New York, NY 10032, USA; Department of Neurology, Vagelos College of Physicians and Surgeons, Columbia University, and the New York Presbyterian Hospital, New York, NY 10032, USA; The Gertrude H. Sergievsky Center, Vagelos College of Physicians and Surgeons, Columbia University, and the New York Presbyterian Hospital, New York, NY 10032, USA; Department of Neurology, Vagelos College of Physicians and Surgeons, Columbia University, and the New York Presbyterian Hospital, New York, NY 10032, USA; The Gertrude H. Sergievsky Center, Vagelos College of Physicians and Surgeons, Columbia University, and the New York Presbyterian Hospital, New York, NY 10032, USA; Department of Neurology, Vagelos College of Physicians and Surgeons, Columbia University, and the New York Presbyterian Hospital, New York, NY 10032, USA; The Gertrude H. Sergievsky Center, Vagelos College of Physicians and Surgeons, Columbia University, and the New York Presbyterian Hospital, New York, NY 10032, USA; Department of Neurology, Vagelos College of Physicians and Surgeons, Columbia University, and the New York Presbyterian Hospital, New York, NY 10032, USA; The Gertrude H. Sergievsky Center, Vagelos College of Physicians and Surgeons, Columbia University, and the New York Presbyterian Hospital, New York, NY 10032, USA; The Gertrude H. Sergievsky Center, Vagelos College of Physicians and Surgeons, Columbia University, and the New York Presbyterian Hospital, New York, NY 10032, USA; Department of Medicine, Vagelos College of Physicians and Surgeons, Columbia University, and the New York Presbyterian Hospital, New York, NY 10032, USA; Department of Anatomy, Physiology and Genetics, Uniformed Services University of the Health Sciences, Bethesda, MD 20814-4799, USA; Department of Anatomy, Physiology and Genetics, Uniformed Services University of the Health Sciences, Bethesda, MD 20814-4799, USA; Department of Neurology, Vagelos College of Physicians and Surgeons, Columbia University, and the New York Presbyterian Hospital, New York, NY 10032, USA; Department of Neurology, Vagelos College of Physicians and Surgeons, Columbia University, and the New York Presbyterian Hospital, New York, NY 10032, USA; The Gertrude H. Sergievsky Center, Vagelos College of Physicians and Surgeons, Columbia University, and the New York Presbyterian Hospital, New York, NY 10032, USA; Department of Medicine, Vagelos College of Physicians and Surgeons, Columbia University, and the New York Presbyterian Hospital, New York, NY 10032, USA; Department of Anatomy, Physiology and Genetics, Uniformed Services University of the Health Sciences, Bethesda, MD 20814-4799, USA; Department of Neurology, Vagelos College of Physicians and Surgeons, Columbia University, and the New York Presbyterian Hospital, New York, NY 10032, USA; Department of Neurology, Vagelos College of Physicians and Surgeons, Columbia University, and the New York Presbyterian Hospital, New York, NY 10032, USA; The Gertrude H. Sergievsky Center, Vagelos College of Physicians and Surgeons, Columbia University, and the New York Presbyterian Hospital, New York, NY 10032, USA

**Keywords:** dystonia, pantothenate-kinase, genetic sequencing, founder effects

## Abstract

Pantothenate-kinase-associated neurodegeneration (PKAN) is a rare, autosomal recessive neurological disorder characterized by the progressive degeneration of specific regions in the brain and is invariably fatal. Several individuals in families affected by PKAN were known to live in an isolated region in a southwestern province of the Dominican Republic and had been previously studied. Forty-six individuals with PKAN in 34 families were evaluated for disease manifestations using the PKAN-Disease Rating Scale and the Leiter-3 Cognitive and Neuropsychological assessment. We completed whole genome sequencing in the 46 affected individuals and their 80 unaffected relatives. Haplotype analysis was used to identify shared genetic patterns among individuals with the mutation to identify common ancestral and founder effects. The classic form of PKAN was observed in 22 individuals with moderate-to-severe oromandibular dystonia and limb dystonia and onset in early childhood. The atypical form was observed in 24 individuals with Parkinsonism, dystonia, cognitive deficits, and later onset of disease. A *PANK2* variant, chr20:3907977: A:G *(c.680A*  *>*  *G, p.Y227C),* was homozygous among 42 affected individuals equally divided by disease form. There were 59 heterozygous carriers of this variant among parents and relatives of the affected individuals. Four individuals from two families were compound heterozygotes for *c.680A*  *>*  *G* and chr20:3918728: C:T (*c.1594C*  *>*  *T).* Haplotype analyses revealed shared patterns across families and of African origin consistent with founder effects for *c.680A*  *>*  *G* and *c.1594C*  *>*  *T*, likely introduced to the island 25–35 generations earlier. The frequency of heterozygous carriers of *c.680A*  *>*  *G* allele among individuals of Dominican ancestry living in New York was 0.18% but was 0.8% among individuals living in the Dominican Republic, significantly higher than the reported frequency for all causal *PANK2* mutations worldwide. This investigation confirmed likely founder mutations in *PANK2* associated with the classic and atypical forms of PKAN in 34 families in an isolated region of the Dominican Republic. Compound heterozygosity was observed in four individuals from two families. The heterozygous frequency of *c.680A*  *>*  *G* was exceptionally high in the Dominican population compared with worldwide data. Founder mutations in such communities offer a unique opportunity to set up relevant, affordable and accessible genetic counselling and screening.

## Teaser

Vardarajan *et al*. used whole genome sequencing in 46 individuals with pantothenate-kinase-associated neurodegeneration and 80 unaffected individuals from 34 families to identify causal mutations in the *PANK2* gene. A homozygous variant was present in most affected individuals. Haplotype analysis confirmed a founder effect and an African ancestral origin.

## Graphical Abstract

**Graphical Abstract:**
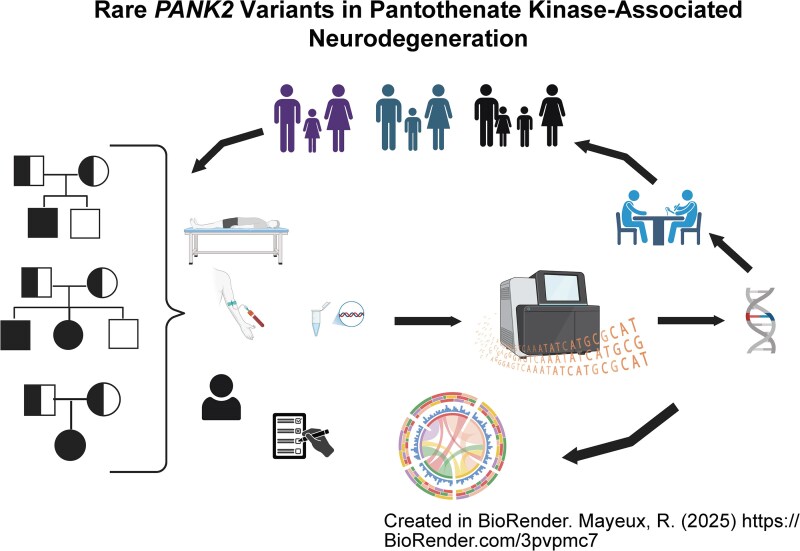

## Introduction

Pantothenate-kinase-associated neurodegeneration (PKAN) is a rare, autosomal recessive neurological disorder characterized by the progressive degeneration in the globus pallidus and surrounding regions of the central nervous system.^1-3^ PKAN is the most common form of neurodegeneration associated with brain iron accumulation (NBIA), a group of clinical disorders characterized by abnormal involuntary movements, alterations in muscle tone, and extrapyramidal signs. Some, but not all these disorders, including PKAN, display characteristic radiographic evidence of iron accumulation in the brain on magnetic resonance imaging, which in PKAN is termed the ‘eye-of-the-tiger’ sign.^3,4^

There are two forms of PKAN.^3^ Classic PKAN begins in early childhood and worsens gradually within the first five years of life. Dystonia is often generalized, beginning in the legs before progressing to the musculature of the mouth, throat and tongue, resulting in progressive dysarthria, dysphagia and eventually loss of speech and swallowing. There may be developmental delay and intellectual impairment, though the latter is difficult to assess due to limited verbal communication. In the classic form of PKAN, individuals experience a rapid course, becoming non-ambulatory by age 10–15 years, and the disease is invariably fatal.^5^ This form may also be associated with retinal degeneration and acanthocytosis.^6^ Atypical PKAN appears later in childhood or early adolescence, usually after the age of 10 years, and progresses more slowly than the classic form. In fact, the loss of ambulation may be delayed up to as much as three decades.^5^ The characteristic manifestations include palilalia, dysarthria, Parkinsonism, personality changes, and gait disorders. Impaired ambulation, mastication and dysphagia compound the overall survival with PKAN regardless of subtype. The phenotype of PKAN can also extend along a continuum without clear delineation between the classic and atypical forms, and there can be variable manifestations of age of onset and semiology.

PKAN is inherited as an autosomal recessive disease, caused by homozygous missense or loss-of-function mutations, or by compound heterozygous missense, completely penetrant, mutations in the *PANK2* gene.^1,3^ While estimates of the frequency are limited globally, PKAN is considered a rare Mendelian disorder. *PANK2* encodes the enzyme pantothenate kinase. Rare variants in this gene alter pantothenate, vitamin B5 metabolism, which is required to produce coenzyme A. Disruption of this enzyme affects energy and lipid metabolism, leading to accumulation of potentially harmful compounds in the brain, including iron.^1^  *PANK2* is the only gene known to be associated with PKAN. The heterozygous frequency of *PANK2* variants has been estimated at less than 0.01% worldwide and varies slightly by ethnic group,^7^ and the incidence of live births with PKAN is between two and three per 1 000 000 (0.0002%) worldwide. There are more than 100 pathogenic or likely pathogenic variants in *PANK2.*^8^

In 2022, we learned of several families affected by PKAN and living in an isolated region in the country, some of whom had been previously studied.^4,6^ Several years earlier, 21 individuals had sequencing of the *PANK2* gene reporting a pathogenic variant, c.680A > G, in the homozygous configuration in 17 of these individuals.^4^ Four years later, the variant was confirmed in this group of individuals and in another eight individuals.^6^ However, colleagues indicated that there might be several more affected individuals in this isolated region of approximately 30 000 individuals, and possibly more in other parts of the country. This meant that the heterozygous frequency of pathogenic *PANK2* alleles in this country might be higher than the worldwide frequency and raised the possibility of a founder mutation. They requested our assistance in setting up a genetic screening programme. We hypothesized that the c.680A > G variant might represent a founder mutation, and thus, the goals of this study were to clinically characterize and genotype all of the affected individuals living in this isolated region.

## Materials and methods

In September 2023, we examined affected individuals with a clinical diagnosis of PKAN and their unaffected family members. We obtained blood for DNA and blood smear to assess acanthocytosis. Most individuals were evaluated at a hospital outpatient facility, but a small number who were unable to travel due to their disability were seen in their homes. A second set of families were evaluated in the capital in Santo Domingo. Brain MRIs had been done previously for diagnostic purposes in 41 of the affected individuals, showing the classic findings of ‘eye-of-the-tiger’ sign, characteristic of PKAN (Table 1).^4,6,9,10^ The MRI protocol included diffusion-weighted and T1- and T2-weighted sequences.

**Table 1:** Demographics of affected individuals with pantothenate-kinase-associated neurodegeneration

	Classic	Atypical	Total
*N*	26	20	46
Age-at-onset (years)	8.5 (3.5)	13.4 (2.6)	10.6 (4.1)
Females (%)	8 (30.8%)	14 (70.0%)	22 (47.8%)
Education (years)	4.9 (2.5)	5.7 (4.1)	5.2 (3.2)
PKAN-DRS			
Total Motor Score	34.7 (13.2)	22.7 (17.5)	29.5 (16.1)
Disability			
Cognition^a^	2.80	2.53	2.67
Speech	3.14	3.10	3.12
Handwriting	3.00	3.00	3.00
Feeding	2.19	2.20	2.20
Eating	1.41	1.58	1.50
Hygiene	2.73	2.76	2.70
Dressing	2.68	2.62	2.60
Walking	4.00	3.33	3.70
School/Work	2.64	2.38	2.50
Behaviours			
Sad	8 (30.8%)	8 (40.0%)	16 (34.8%)
Anxiety	11 (42.3%)	6 (30.0%)	17 (36.9%)
Suicide	0	0	0
Aggressive	11 (42.3%)	9 (45.0%)	20 (43.5%)
Irritable	12 (46.2%)	8 (40.0%)	20 (43.5%)
Obsessions	4 (15.4%)	4 (20.0%)	8 (17.4%)
Hallucinations	0	0	0
Leiter-3^b^			
Nonverbal IQ^b^	50.8	55.9	53.1

PKAN-DRS Motor Score is the sum of all of the motor manifestations: including bradykinesia, rigidity, finger tapping, let agility, gait, dystonia, chorea, spasticity, tremor, and oculomotor dysfunction. Disability was ranked on a Likert scale from 0 to 4, with 0 representing the absence and 4 representing the most severe. The table shows the mean score per group. Behaviour was scored as present or absent.

Leiter-3.

^a^Seven individuals were unable to be tested, and it was not possible to assess cognition.

^b^The nonverbal IQ is standardized using norms representing the general population in terms of ethnic group, race, sex and age, *X* + 100, SD = 15.

Our protocol was approved by the respective agencies in the Dominican Republic and the Institutional Review Board at Columbia University in accordance with the Declaration of Helsinki. All participating individuals or their designated authorized representative gave informed consent. Our assessments included a standardized medical history, medications used, a detailed family history with pedigree construction, neuropsychological testing, and videotaping of a structured neurological examination that included the PKAN-Disease Rating Scale.^11^ With the help of the family, we attempted to determine the age-at-onset of symptoms by asking when they first noticed any sign or symptom possibly related to PKAN. We also asked when the affected individuals were last considered symptom free.

*PKAN-Disease Rating Scale (PKAN-DRS)*:^11^ We assessed each individual in two phases. The first section rated cognition, behaviour and disability using a modified Likert Scale from 0 (no impairment) to 4 (severe impairment). The behavioural assessment queried the presence or absence of sadness, anxiety, suicidal thoughts, aggression, irritability, obsessions, and hallucinations, but not the severity of these symptoms. The disability assessment included speech, handwriting, feeding, eating, swallowing, hygiene, dressing, walking, schoolwork and vision. The second section rated motor manifestations using a similar Likert Scale approach and included Parkinsonism (bradykinesia, rigidity, finger tapping, leg agility, freezing of gait, postural or truncal stability, and resting tremor), Dystonia (upper and lower face, eyes, jaw, tongue, neck, arms, trunk and legs), speech, gait, chorea, spasticity, action or postural tremor, and oculomotor dysfunction. The second section was videotaped for consensus review and scoring by a group of independent movement disorder specialists at Columbia University.

*Leiter-3 Nonverbal Cognitive and Neuropsychological assessment*:^12^ The Leiter-3 is a reliable and valid nonverbal test developed for individuals aged 3 to 75 years that uses standardized scores based on a normative sample reflecting the general population in terms of ethnicity, race, gender, and age. The measure is specifically designed for use in individuals with cognitive, speech or hearing dysfunction, motor impairment, as well as individuals whose dominant language is not English. The measure uses a refined block-and-frame format with lightweight blocks and foam manipulatives to reduce the effects of impaired motor function, as well as visual stimuli (patterns, sequence, and illustrations) that do not require the use of language. For this study, two physicians (F.B.A. and F.A.R.) were trained in the application of the Leiter-3 assessment and tested all individuals included in the study.

*Whole blood and DNA extraction*: A blood sample was obtained for DNA extraction and collected from all individuals providing consent, which included both affected and unaffected family members. Blood was shipped overnight to Columbia University in New York City, where DNA was extracted and stored. An additional sample was sent to CEDIMAT in Santo Domingo to measure acanthocytosis by blood smear and was examined under a microscope to count the percentage of abnormally shaped cells.

*Data Analysis*: Continuous variables such as age, years of education, and summary scores from phenotype scales were analysed using IBM SPSS Statistics to determine means and standard deviations. For dichotomous variables, we used chi-square module on SPSS.

*Whole Genome Sequencing*: To identify variants including single-nucleotide variants (SNVs), indels, complex structural variation, including insertions, deletions, copy number variation and tandem repeats, all affected individuals and their family members underwent whole genome sequencing PCR-free DNA library preparation was conducted by automation robotics ligation-based workflow. Quality-controlled libraries were sequenced on the Illumina NovaSeq 6000 or X Plus at the Uniformed Services University of the Health Sciences in Bethesda, MD. Samples were sequenced to a depth of ≥30 × mean coverage. Raw reads were aligned to the human reference genome build 38 using the Burrows–Wheeler Aligner.^13^ Quality control of the sequencing data and variant calling was done using Genome Analysis Toolkit (GATK).^14^ Called variants were further QCed using VariantRecalibration in GATK.

*Sequence Analyses*: Variants passing the Variant Quality Score Recalibration threshold were filtered for sample missingness (>2%), depth of coverage DP > 10 and genotype quality-GQ > 20. We annotated high-quality variants using ANNOVAR. Specifically, variants were annotated for population-level frequency using Genome Aggregation Database (gnomAD),^15^ TOPMed and Database of Structural Variation (DGV),^16^  *in silico* function using Ensembl Variant Effect Predictor (VEP)^17^ and variant conservation using Combined Annotation Dependent Depletion score (CADD)^18,^ as well as variant-disease association from Online Mendelian Inheritance in Man (OMIM),^19^ Human Gene Mutation Database (HGMD)^20^ and ClinVar.^21^ PKAN-associated variants were expected to be significantly enriched in this cohort. We tested enrichment of putatively causal coding variants in the *PANK2* gene in the affected individuals in the cohort. Additionally, we tested the association of single-nucleotide variants in the genome with disease phenotypes, classic and atypical forms and with age-at-onset of symptoms related to PKAN.

Local ancestry was estimated using common variants defined with a minor allele frequency of less than 5% (minor allele frequency [MAF] ≥ 5%) in the 5 MB region flanking the *PANK2* gene. Haplotypes of affected and unaffected individuals were phased using Shapeit2 software.^22,23^ Reference haplotypes from three continental ancestries- non-Hispanic Whites (CEU), African (Yoruban) and native Americans (Pima, Sura, Maya and Incas) were obtained as previously described. Local ancestry proportions for each single-nucleotide polymorphism (SNP) were determined using RFMix software,^24^ and the haplotype was assigned one of the three ancestries if at least 90% of the variants within the gene were of a specific ancestry.

## Results

We evaluated 126 individuals from 35 families, including 46 affected individuals equally distributed between the classic and atypical forms (Table 1). The mean age of the affected individuals was 26.3 (± 10.4 SD) years, and the mean age at onset was 10.7 (± 3.8 SD) years (ranging from 2 to 20 years). However, symptom onset was uncertain for five individuals. For those classified as having the classic form, the mean age at onset was 8.1 (± 2.2 SD) years, while for those with the atypical form, it was 13.6 (± 2.7 SD) years. Girls or young women represented 51.1% of the affected individuals. On average, affected individuals had 5 years of education compared to 9 years among the unaffected family members of similar age. Among the 75 family members, 49 (65%) were parents of the affected individuals, 5 were unaffected siblings, and the remaining 11 were unaffected more distant relatives.

Phenotypes. Among the 46 affected individuals, dystonia was the most prominent manifestation, followed by Parkinsonism (Supplementary Table 1). Generalized dystonia was seen in most affected individuals. Facial dystonia was particularly prominent, either exclusively oromandibular or disproportionally oromandibular, with or without moderate blepharospasm. Mild to moderate bradykinesia was apparent in all affected individuals, but worse in those with prominent dystonia. The classic form of PKAN,^3^ had onset between the ages of 3 and 10 years with rapid progression of symptoms, and was present in 54% of affected individuals. Clinically, these patients exhibited severe generalized dystonia often involving the oromandibular and buccolingual regions, resulting in dysarthria and dysphagia. The atypical form of PKAN,^3^ with onset after age 10 years in adolescence or adulthood, was present in 46% of affected individuals. This form was associated with a more gradual progression based on family report. We observed Parkinsonism without tremor, namely bradykinesia and some rigidity, in most individuals with atypical PKAN. Two brothers carrying *PANK2* mutations, however, exhibited not only asymmetrical mild bradykinesia and rigidity, but also mild and intermittent resting tremor (i.e. tremor >1 cm but <3 cm in maximal amplitude) as well as shuffling gait, festination and freezing. Visual deficits were suspected in a few subjects with either classic or atypical PKAN, but whether they had pigmentary retinopathy could not be readily established. In four affected individuals (8.7%), we could not determine the type of PKAN, but most had a later age at onset, suggesting the atypical form. Acanthocytosis^6^ was present in 17 (39.5%) of the 43 affected individuals with PKAN who were tested.

Using the PKAN-DRS,^11^ we identified a variety of behavioural symptoms in the cohort. Aggressive and irritable behaviour was present in 20 (43.5%) individuals and was the most frequent symptom. Other symptoms reported were sadness in 16 individuals (34.7%), anxiety in 17 (36.9%), and obsessiveness in four (8.7%). No behavioural symptoms were reported in six (13%) individuals. The PKAN-DRS also ranks the total number of behaviours present in each individual. Among the 40 individuals with behavioural symptoms, 18 (45%) had only a single symptom, 15 (37.5%) had two to three symptoms and seven (17.5%) had four or more. There were no statistically significant differences in the behaviours between the Classic and Atypical forms (Table 1).

Data acquired on the Leiter-3 scale determined the general cognitive functioning. Assessment of cognitive ability was not possible in 5 (11%) of the sample due to severe motor or cognitive limitations to perform and/or a poor understanding of instructions. The measure was completed in 41 individuals ranging in age from 7 to 54 years. The mean overall nonverbal IQ was moderately impaired (X̅ = 53.39, SD = 14.0; ranging from 30 to 81) with performance ranging from severe impairment to low average, and did not significantly differ between individuals with the classic or atypical phenotype: 51.8 (SD = 15.8) versus 55.3 (SD = 11.6), respectively. Some variability in individual subtest performance was driven by limited physical movements due to dystonia and by fatigue.

**Figure 1:**
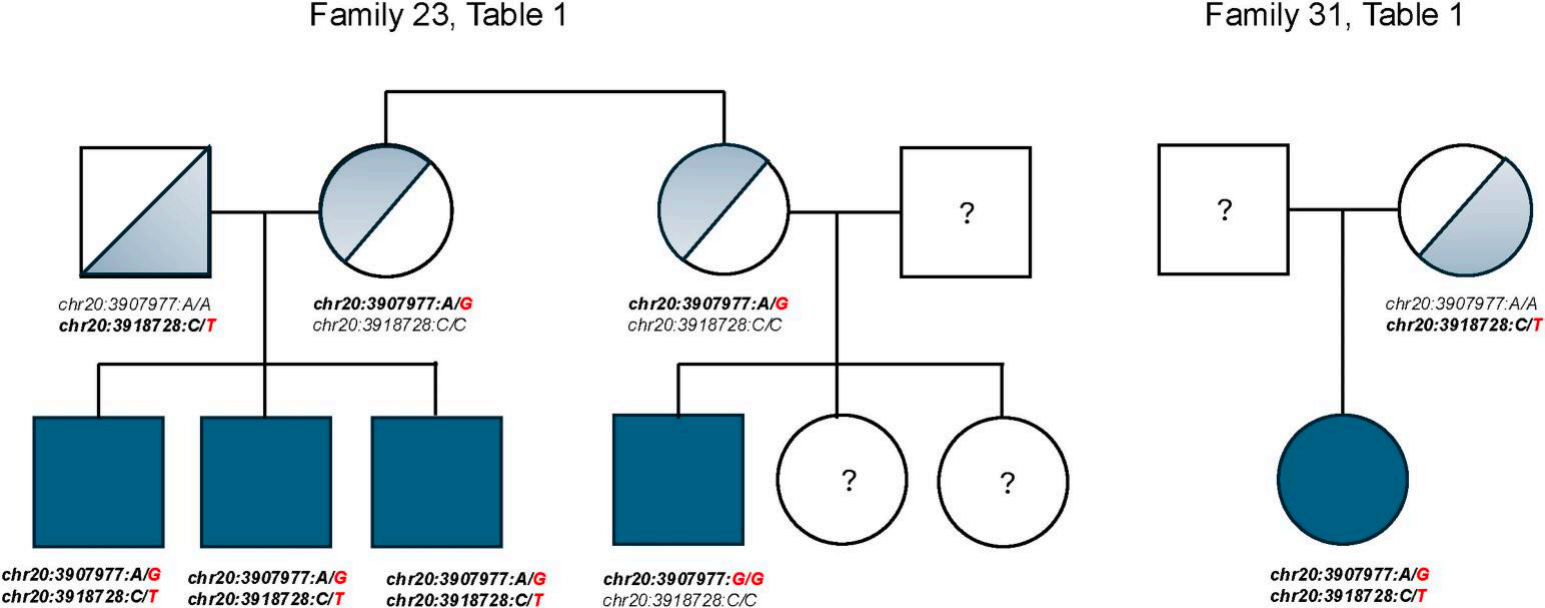
***PANK2* compound heterozygous variants in two families.**  *PANK2:* chr20:3907977:A:G *(c.680A > G, p.Y227C)* and chr20:3918728:C:T (c.1594C > T, *p.R422W).*

Whole genome sequencing. A pathogenic variant, *PANK2* chr20:3907977:A:G *(c.680A*  *>*  *G, or p.Y227C* on Transcript: ENST00000316562.9) was present in the homozygous configuration among 42 affected individuals from 34 families. There were 59 heterozygous carriers of this variant, all of whom were either parents or other relatives of the affected individuals. Interestingly, three affected individuals from one family, and a single affected individual from a second family were *PANK2* compound heterozygous: *c.680A*  *>*  *G* and chr20:3918728:C:T (*c.1594C*  *>*  *T or p.R422W on Transcript: ENST00000610179.7)*. This second missense variant has a Combined Annotation Dependent Depletion Score (CADD) of 26.4 that defines it as damaging^25^ and was extremely rare in gnomAD (MAF = 0.0000037) or other population databases. Three of the four were brothers from a single family (Fig. 1). The other individual was an offspring of a *c.1594C*  *>*  *T* heterozygous carrier. An earlier report of this family was published as an abstract.^26^ All pathological variants were confirmed in a Clinical Laboratory Improvement Amendments (CLIA) laboratory, enabling return results to physicians and their patients and families. Whole genome sequencing was used to identify possible differences in specific disease phenotypes, but no single-nucleotide variants in the region surrounding *PANK2*, nor in any other part of the genome, to explain the phenotypes (classic versus atypical) or age-at-onset of symptoms.

We used existing data from several sources to determine the heterozygous carrier frequency of the *c.680A*  *>*  *G*, *PANK2* variant. Whole exome sequencing data from the Institute of Genomic Medicine at Columbia University were reviewed in 7127 individuals of Hispanic ancestry from the Dominican Republic and living in the communities of Washington Heights, Inwood and Hamilton Heights in New York City. None had PKAN and were participating in multiple genetic studies. We found 13 (0.18%) individuals were heterozygous carriers for *c.680A*  *>*  *G* allele. An additional 6414 individuals of mixed Hispanic and African ancestry were also assessed. Ten (0.15%) were heterozygous carriers of the *c.680A*  *>*  *G* allele. We also had previously acquired whole genome sequencing data from 2120 elderly individuals from 487 families without PKAN living in the Dominican Republic, participating in a study of familial Alzheimer’s disease. Selecting one individual from each family, we identified four unrelated heterozygous carriers of the *PANK2 c.680A*  *>*  *G* allele, yielding a carrier frequency of 0.82% which is approximately an 80-fold increase compared to the worldwide frequency of any pathologic or likely pathologic *PANK2* variants.^7^ At this observed frequency, we would expect the incidence rate of PKAN in the Dominican Republic to be one per 15 000 births, which is 100 times more prevalent than global estimates in African Americans and 25–32 times more prevalent than in non-Hispanic white and Hispanic populations.^7^ There were no heterozygous carriers of the *c.1594C*  *>*  *T* variant allele in *PANK2* in any of these three cohorts, nor was it reported in the Genome Aggregation Database.^15^

The high frequency of the *PANK2 c.680A*  *>*  *G* allele in the Dominican Republic and among individuals of Dominican ancestry prompted us to investigate the ancestral origin of this mutation in this group of affected individuals and families. We used 15 common variants within the *PANK2* gene to determine the ancestry of the haplotype of mutation carriers and non-carriers. Among homozygous *PANK2 c.680A*  *>*  *G* allele affected individuals, 80.5% inherited haplotypes from an African ancestry on both chromosomes. The remaining affected individuals inherited at least one chromosome from an African background. Similarly, all compound heterozygous PKAN individuals also had an African haplotype on both chromosomes. Remarkably, among relatives of affected individuals with PKAN who were heterozygous for *PANK2 c.680A*  *>*  *G* allele, 89.3% had at least one African ancestral haplotype within the *PANK2* gene.

Assuming that the *PANK2 c.680A*  *>*  *G* allele represents a founder mutation, we estimated the age of the variant allele using the haplotype described above in the 5 MB region flanking *PANK2* gene. We used the *Gamma method,*^27^ which is based on ancestral segment lengths, to determine the genetic length of ancestral haplotypes shared between individuals carrying the mutation. The resulting estimate suggested that the homozygous founder *PANK2 c.680A*  *>*  *G* allele was likely introduced to the island 35.9 (95% CI, 28.9–44.7) generations earlier.^27^

## Discussion

In an isolated region in the Dominican Republic, we identified 42 affected individuals homozygous for the chromosome 20 *c.680A*  *>*  *G* variant allele in *PANK2.* While *PANK2* had previously been sequenced in some individuals, this was the first whole genome assessment of these individuals and their family members.^4,6^ Four additional affected individuals, heterozygous for this variant allele and a second variant allele on chromosome 20 *c.1594C*  *>*  *T,* were also identified among a total of 35 families. Slightly more individuals had the classic form than the atypical form in these families. Many affected individuals had previously been reported to have the eye-of-the-tiger sign^4,9,10^ on brain MRI, showing lesions in the globus pallidus, but not all had been sequenced. Using genetic data from independent studies that included individuals of Hispanic ancestry from the Dominican Republic who were free of PKAN, we found the carrier frequency of the *c.680A*  *>*  *G* variant in *PANK2* to range from 0.18% to 0.82%, which was significantly higher than all known mutations in *PANK 2* worldwide combined.^7^

The classic form of PKAN was significantly more frequent in boys and young men than in girls and young women in this group of patients, but these sex differences were reversed for the atypical form of the disease. In general, the inheritance pattern is the same for women and men.^28^ However, as we have observed, individual studies may report sex differences within their cohort of patients, reflecting the study protocol or patient availability.^29-32^

Movement disorders are prominent features in PKAN, with dystonia and Parkinsonism among the most frequently reported manifestations in both classic and atypical forms of PKAN.^2,3^ Our two identified *PANK2* mutations have essentially been found in patients of Dominican Republic descent, and none of the 46 individuals from this cohort presented with a neurological phenotype that differed from that reported in patients carrying more common *PANK2* mutations.^2,3^ This is in keeping with genotype-phenotype correlation studies showing that among over 100 distinct *PANK2* mutations, none was associated with a unique neurological phenotype.^33,34^

Consistent with previous findings,^2,3^ dystonia in classic PKAN was often generalized. While limb dystonia was quite disabling, limiting the ability of individuals to perform daily tasks such as walking or using their hands, the most dramatic features were oromandibular dystonia, characterized by involuntary tongue protrusion and jaw opening with severe drooling; one individual had prominent opisthotonus. In all affected individuals with oromandibular dystonia, it was never focal but rather associated with limb dystonia and additional cranial dystonia such as torticollis and, to a lesser extent, blepharospasm. In most, oromandibular dystonia was associated with severe dysphonia and dysarthria,^35-37^ rendering speech unintelligible and impairing swallowing, which precluded feeding by mouth. Although severe cases of tongue protrusion dystonia may lead to self-injurious behaviour, such as tongue and lip biting,^37^ this was not observed in this cohort. In this cohort, several individuals used a small towel to absorb excess saliva, and one individual placed a toothbrush in her mouth to lessen jaw opening and tongue protrusion.

In addition to dystonia, Parkinsonism was present but generally milder than dystonia among individuals with classic PKAN. Mild bradykinesia was commonly observed in our classic PKAN cases, adding to the functional impairments caused by dystonia. Mild rigidity was present in some subjects. Among the other neurological features associated with PKAN,^2,3^ we did not observe any classic cases with chorea or upper motor neuron signs. However, some subjects exhibited severe, painless muscle atrophy, particularly in the lower limbs, without evidence of fasciculations. This atrophy appeared to exceed what would be expected from disuse alone and contributed to marked weakness and restricted mobility. Additional diagnostic testing, including electromyography and nerve conduction velocity, was unavailable but could be considered in a next-step study. A review of the literature on PKAN, only rarely mentions muscle atrophy as part of the clinical presentation in PKAN patients.^38^

Atypical presentations of PKAN add to its clinical complexity. For instance, in some cases, affected individuals, especially with late onset, exhibit Parkinsonism as a predominant feature rather than dystonia.^2,3^ The systematic review from Botsford *et al*.^39^ analysed 11 studies published between 1981 and 2018 that described PKAN patients presenting with Parkinsonism, providing a valuable analysis about the clinical presentation of this association. All publications reported features consistent with atypical Parkinsonism, including lack of asymmetry, coexisting dystonia, and poor levodopa responsiveness. However, one patient with atypical PKAN with Parkinsonism was reported to show improvement in tremor following levodopa administration.^40^ Notably, two brothers with late-onset PKAN exemplify this atypical presentation, as both presented with marked Parkinsonism, including freezing of gait, a symptom associated with Parkinson's disease or atypical Parkinsonism as well as PKAN; one individual had no associated dystonia at 23 years’ disease duration. These two individuals underscore the phenotypic heterogeneity of PKAN, with certain genetic or environmental modifiers potentially influencing the expression of either dystonia or Parkinsonism. While both individuals were homozygous for the *c.680A*  *>*  *G* variant allele in *PANK2*, they were distinct from other individuals with atypical PKAN. A review of their sequencing data yielded no evidence of mutations in *PRKN* or other known genes,^41^ suggesting that this isolated Parkinsonism, though unusual, is likely part of the PKAN neurological spectrum rather than a reflection of concomitant disorders.

Although not observed in the families reported here, Rohani *et al*.^42^ reported three individuals with atypical PKAN who presented with a tremor-dominant form of the disease, which followed a relatively benign course. Among these individuals, one had dystonic tremor and two had Parkinsonian tremor. All three patients had homozygous mutations in the *PANK2* gene and displayed the characteristic ‘eye-of-the-tiger’ sign on brain MRI. This underscores PKAN as a potential cause of tremor and emphasizes the need to consider the diagnosis of PKAN even in patients initially diagnosed with essential, dystonic, or Parkinsonian tremor.

The cognitive phenotype in PKAN can be quite variable, with performance ranging within the impaired range to within the normative average range. These results have greatly depended upon how conceptual abilities are measured, given physical and potential language limitations. Deficits in specific cognitive domains, including attention, executive functioning, and verbal and visual learning, have also been reported in PKAN.^43^ Multifactorial contributions likely impacted cognitive performance within the current group of individuals examined here. Aside from genetic influence on brain development, there are likely socioeconomic and physical limitations that affect intellectual development. These contributors will need to be further examined to better understand genotype–phenotype correlations.

Several neurological disorders, including PKAN, have been associated with acanthocytosis and are also genetically defined diseases characterized by progressive degeneration of the basal ganglia and neuromuscular manifestations often accompanied by elevated levels of creatine kinase.^44,45^ Autosomal recessive chorea-acanthocytosis and X-linked McLeod syndrome all share psychiatric symptoms, cognitive impairment, peripheral neuropathy, myopathy and epilepsy. As is present in PKAN, affected individuals may also have facial and oromandibular dystonia, tics, Parkinsonism and postural tremor. Chorea-acanthocytosis has been associated with loss-of-function variants in the *VPS13A* gene.^46^

Pantothenate-kinase-associated neurodegeneration (PKAN, OMIM 234200), is the most common form of NBIA. It is an autosomal recessive disorder resulting from rare pathogenic mutations in the *PANK2* gene (OMIM 606157).^3,34,47,48^ The Genome Aggregation Database has 66 variants listed as likely pathogenic or pathogenic,^15^ but there are several hundred mutations described. Compound heterozygotes in *PANK2* are also frequently observed. It has been estimated that the incidence rate of PKAN is two in every million live births,^7^ but by using the heterozygous frequency we observed in this study, the incidence rate would be much higher among individuals from the Dominican Republic.

Biallelic variants in *PANK2* alter the function of mitochondrial pantothenate kinase 2, reducing coenzyme A (CoA) metabolism primarily in the basal ganglia, especially in the globus pallidus.^7,49^ Normally, PANK2 activates and maintains mitochondrial acyl carrier protein.^2^ Once activated, this protein provides support for fatty acid synthesis, iron-sulphur cluster biogenesis and electron transport. Variants in *PANK2* disrupt the function of pantothenate kinase 2 in the mitochondria, resulting in defective CoA biosynthesis and the subsequent accumulation of iron and toxic secondary metabolites downstream of this pathway.^50^ While iron overload may contribute to the symptoms of PKAN, it is more likely a complex interaction between lipid metabolism, mitochondrial dysfunction and autophagy.^51^

The cohort described here may be one of the largest groups of affected individuals with the same mutation in the homozygous configuration. The origin of the *c.680A*  *>*  *G* variant allele in *PANK2* is also of great interest. Haplotype analysis, in which DNA sequence was examined around a specific variant of interest on chromosome 20, was used to identify shared genetic markers among affected individuals. This finding allowed us to infer a common ancestry or founder effect in order to track the inheritance of the mutation through generations.^52^ Individuals with a founder mutation will often share a large region of identical DNA around the mutated gene, the haplotype, which can be traced back to the original founder. Such ‘founder mutations’ are most readily identified in populations with a high degree of genetic homogeneity, like isolated communities^53,^ such as those in the Barahona Province (Supplementary Fig. 1).

There are some limitations to this report. We are aware that several additional families reside in nearby villages. They may have additional variants that need to be identified. The genome sequencing analyses were focused on causal single-nucleotide variants. Further analyses might facilitate identification of putative modifiers of phenotypic variation, such as age-at-onset or individual neurological manifestations. We did not fully assess the effects of the environment on these affected individuals.

Finally, the genetic architecture of an isolated community can be influenced by the geographical region, population migration, selection pressure, and cultural practices, which influence the unique variants present in the specific population. The part of the country we visited is relatively isolated because it is surrounded by mountains and separated by a river. The application of genomics in community settings, such as this one in the Dominican Republic, provides an opportunity to identify founder variants associated with human disease. The identification and characterization of founder variants can have implications for understanding the causes of the disease, and in implementing social policies such as genetic testing, enabling precise diagnosis and prevention in an attempt towards precision medicine.^54,55^ This report summarizes an initial step in mobilizing genetic testing and counselling for these families over the next few years.

## Supplementary Material

fcaf286_Supplementary_Data

## Data Availability

Anonymous clinical and genetic data from this study are available by email request to the authors at Columbia University. The data are held on a secure website that also provides a Qualtrics form to be filled out by the investigators with contact information, title of the project, type of data requested, brief description of rationale for the request, co-authors, research question to be addressed, and approval by the institutional ethical review board.
