# Supplementary material for: Rare PANK2 variants and pantothenate-kinase-associated neurodegeneration in the Dominican Republic
Source: Brain Commun. 2025 Aug 4;7(4):fcaf286. doi: 10.1093/braincomms/fcaf286 (PMC12342184; doi:10.1093/braincomms/fcaf286)

Supplementary Table 1. Detailed Phenotypic Findings of 46 Individuals with PKAN.
Y= neurological manifestation is present. MRI + indicates that previously acquired brain MRI showed the classical eye-of-the-tiger sign.

Family	Person	Sex	Disease type	Range Age at Onset	Range Current Age	Parkinsonism	Dystonia	Speech impairment	Tremor	MRI	*c.680A>G*	*c.1594C>T*
1	Proband	M	Classic	2-10	13-19	Y	Y	Y	N	+	1/1	0/0
2	Proband	M	Classic	2-10	34-40	Y	Y	Y	N	+	1/1	0/0
3	Proband	F	Atypical	11-20	27-33	Y	Y	Y	N	+	1/1	0/0
4	Proband	M	Atypical	11-20	41-47	Y	Y	Unknown	Rest	+	1/1	0/0
5	Proband	F	Atypical	11-20	13-19	Y	Y	Y	N	+	1/1	0/0
5	Cousin of Proband	M	Classic	2-10	13-19	Y	Y	Y	N	-	1/1	0/0
6	Proband	M	Classic	2-10	27-33	Y	Y	Y	Action	+	1/1	0/0
6	Sister of Proband	F	Classic	2-10	20-26	Y	Y	Y	N	+	1/1	0/0
7	Proband	F	Classic	2-10	13-19	Y	Y	Unknown	N	+	1/1	0/0
8	Proband	M	Classic	2-10	20-26	Y	Y	Y	N	+	1/1	0/0
9	Proband	M	Classic	2-10	20-26	Y	Y	Y	N	+	1/1	0/0
10	Proband	F	Atypical	11-20	20-26	Y	Y	Y	N	+	1/1	0/0
10	Brother of Proband	M	Atypical	11-20	13-19	Y	Y	Y	N	-	1/1	0/0
11	Proband	M	Classic	2-10	20-26	Y	Y	Y	N	+	1/1	0/0
11	Cousin of Proband	F	Classic	2-10	41-47	Y	Y	Y	N	-	1/1	0/0

12	Proband	M	Classic	2-10	20-26	Y	Y	Y	N	+	1/1	0/0
13	Proband	M	Classic	2-10	20-26	Y	Y	Y	N	+	1/1	0/0
14	Proband	F	Atypical	11-20	20-26	Y	Y	Y	N	+	1/1	0/0
15	Proband	F	Atypical	11-20	41-47	Y	Y	Y	N	+	1/1	0/0
16	Proband	F	Classic	2-10	27-33	Y	Y	Y	N	+	1/1	0/0
16	Brother of Proband	M	Atypical	11-20	41-47	Y	Y	Y	N	-	1/1	0/0
17	Proband	F	Atypical	11-20	13-19	Y	Y	Y	N	+	1/1	0/0
18	Proband	F	Classic	2-10	27-33	Y	Y	Y	N	+	1/1	0/0
18	Sister of Proband	F	Classic	2-10	27-33	Y	Y	Y	N	+	1/1	0/0
19	Proband	F	Classic	2-10	41-47	Y	Y	Y	N	+	1/1	0/0
20	Proband	F	Atypical	11-20	27-33	Y	Y	Y	N	+	1/1	0/0
21	Proband	F	Atypical	11-20	27-33	Y	Y	Y	N	+	1/1	0/0
21	Sister of Proband	F	Classic	12-10	20-26	Y	Y	Y	N	+	1/1	0/0
22	Proband	F	Classic	2-10	20-26	Y	Y	Y	N	+	1/1	0/0
23	Proband	F	Atypical	11-20	20-26	Y	Y	Y	N	+	1/1	0/0
23	Brother of Proband	M	Atypical	11-20	41-47	Y	N	Y	Rest	+	0/1	0/1
23	Brother of Proband	M	Classic	2-10	41-47	Y	Y	Y	Rest/Action	+	0/1	0/1

24	Proband	M	Atypical	11-20	41-47	Y	Y	Y	Rest/Action	+	0/1	0/1
24	Cousin of Proband	F	Classic	2-10	34-40	Y	Y	Y	Action	+	1/1	0/0
25	Proband	F	Atypical	11-20	27-33	Y	Y	Y	Action (fine)	+	1/1	0/0
26	Proband	M	Classic	2-10	13-19	Y	Y	Y	N	+	1/1	0/0
26	Brother of Proband	M	Classic	2-10	13-19	Y	Y	Y	N	+	1/1	0/0
27	Proband	F	Atypical	11-20	27-33	Y	Y	Y	N	+	1/1	0/0
27	Sister of Proband	F	Atypical	11-20	13-19	Y	Y	Y	N	+	1/1	0/0
28	Proband	F	Atypical	11-20	27-33	Y	Y	Y	N	+	1/1	0/0
29	Proband	F	Classic	11-20	27-33	Y	Y	Y	Action (high amplitude)	+	1/1	0/0
30	Proband	M	Atypical	11-20	20-26	Y	Y	Y	N	+	1/1	0/0
31	Proband	M	Atypical	11-20	27-33	Y	Y	Y	N	+	0/1	0/1
32	Proband	M	Classic	2-10	6-12	Y	Y	Y	N	+	1/1	0/0
33	Proband	M	Atypical	11-20	13-19	Y	Y	Y	N	+	1/1	0/0
34	Proband	M	Classic	2-10	13-19	Y	Y	Y	N	+	1/1	0/0

Supplemental Figure 1. Dominican Republic

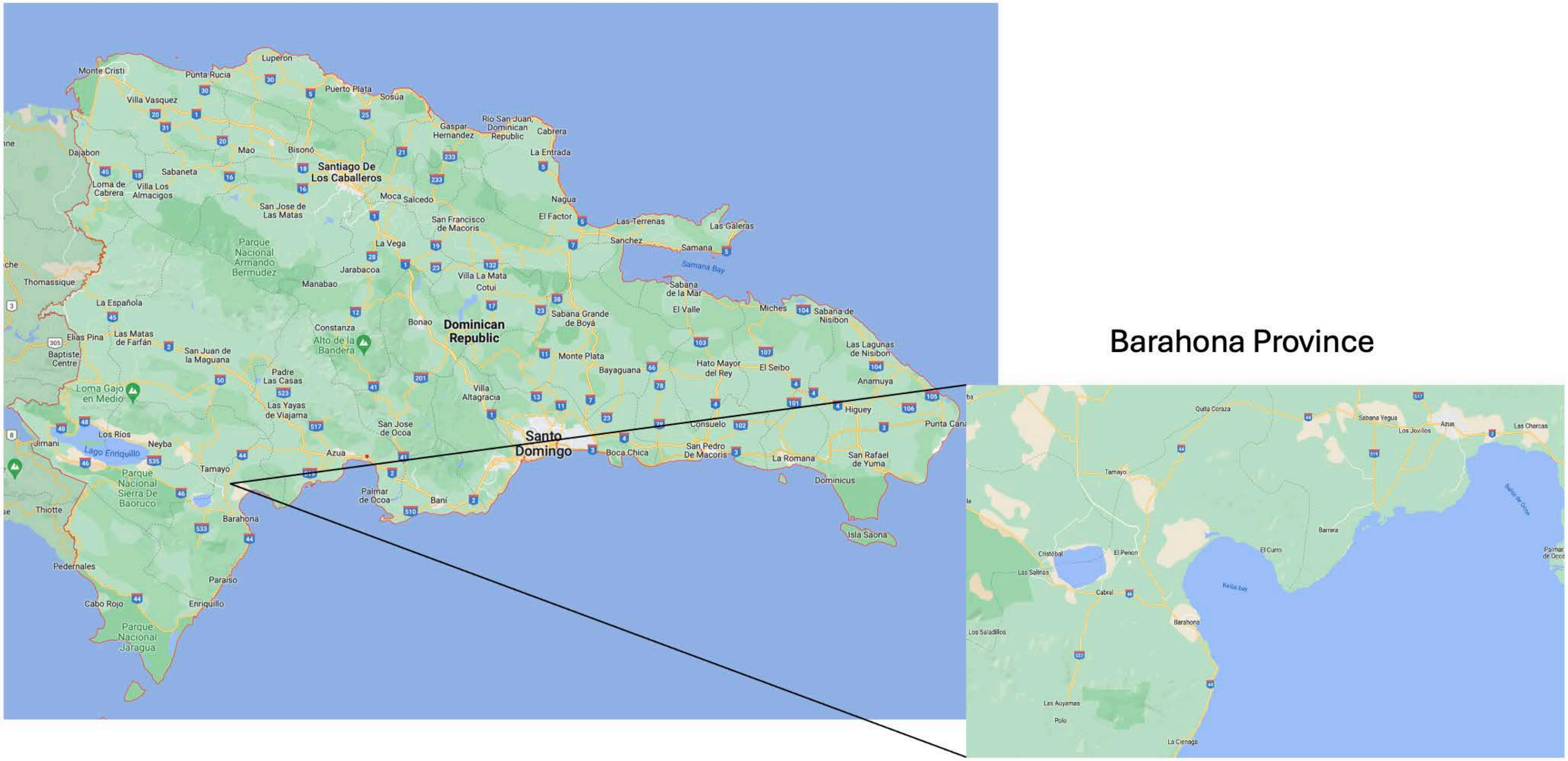

Supplement: fcaf286_Supplementary_Data [file fcaf286_supplementary_data.pdf]
